# Parkinson’s disease quality of life at 12 months comparing invasive device-aided therapy with oral treatment

**DOI:** 10.1038/s41531-025-01093-x

**Published:** 2025-08-11

**Authors:** Adolfo Ramirez-Zamora, Michael S. Okun, Pavnit Kukreja, Wei Hu

**Affiliations:** 1 Department of Neurology, Norman Fixel Institute for Neurological Diseases, University of Florida, Gainesville, FL USA; 2 AbbVie Inc., North Chicago, IL USA

**Keywords:** Parkinson's disease, Quality of life

## Abstract

Real-world impact of device-aided therapy on quality of life (QoL) in people with Parkinson’s remains unknown. This large, retrospective, observational study assessed QoL in people transitioning to device-aided therapy (deep brain stimulation or carbidopa-levodopa enteral suspension) vs. those continuing oral medications. Cohorts were matched based on clinical/demographic characteristics and device-aided therapy eligibility. Primary and secondary outcomes included change from baseline (CFB) to month 12 in 39-item Parkinson’s Disease Questionnaire (PDQ-39) and Unified Parkinson’s Disease Rating Scale (UPDRS) scores, respectively. Of 608 people (oral therapy [*n* = 295]; device-aided therapy [*n* = 313]), most were male, White, and aged ≥ 60 years. Positive CFB to month 12 in PDQ-39 was significantly greater for device-aided therapy vs. oral therapy (–5.0 [95% CI, –5.9 to –4.2] vs. 0.9 [0.3–1.5]; *p* < 0.001). UPDRS II-IV scores improved for the device-aided therapy group. People transitioning to device-aided therapy experienced clinically meaningful QoL improvements.

## Introduction

The current focus of Parkinson’s disease (PD) treatment is symptomatic, primarily targeting symptom management to positively impact quality of life (QoL)^1^. Oral levodopa remains the gold standard of PD treatment^2^. However, the reliability of oral levodopa to adequately control PD symptoms will progressively decrease and dosage timing and frequency may change^3,4^. Pulsatile dopaminergic stimulation, which is associated with the short half-life of oral levodopa, irregular gastric motility, and diminished brain buffering capacity, is thought to contribute to motor symptom fluctuations and complications^3–5^.

As the therapeutic window for the benefits of oral levodopa diminishes, people with PD commonly experience worsening of motor and nonmotor symptoms that can negatively impact QoL^6–8^. In people with moderate to advanced disease, PD symptom management frequently involves complex adaptations to treatment regimens, an increasing pill burden, and reduced adherence to therapy^9,10^.

These challenges can potentially be addressed by device-aided therapies (eg, carbidopa-levodopa enteral suspension [CLES] or deep brain stimulation [DBS]). Despite the well-established efficacy of device-aided therapies for reducing “Off” time and improving motor and nonmotor symptoms in people with advanced disease^11–15^, the use of device-aided therapy remains low^16,17^. Additionally, there are limited data assessing long-term clinical outcomes and QoL in real-world, pragmatic, nonrandomized settings comparing patients who remain on oral therapies vs those who transition to device-aided treatments.

At the University of Florida Norman Fixel Institute for Neurological Diseases, data from people with PD have been collected since 2002 in the INFORM database. Rating scales for specific disorders, medications, QoL questionnaires, and mood scales were collected at every clinic visit.

The objective of this cohort analysis was to compare the change over 12 months in QoL, disease burden, and clinical outcomes in a large cohort of people with advanced PD who either remained on oral therapies or who switched to device-aided therapies.

## Results

### Disposition of people with PD and their characteristics

**Fig. 1:**
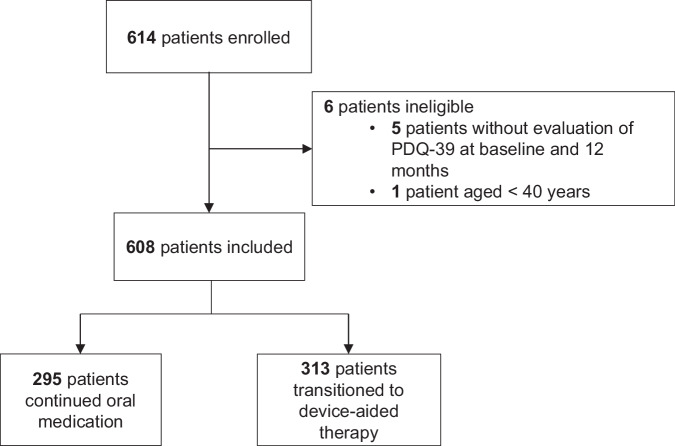
Patient disposition. PDQ-39 39-item Parkinson’s Disease Questionnaire.

Between January 1, 2012, and December 31, 2021, 614 people from the University of Florida clinical database (INFORM) were enrolled, of which six were excluded (five did not have sufficient data, and one was aged <40 years) and 608 met the eligibility criteria (continued oral therapy, *n* = 295; transitioned to device-aided therapy, *n* = 313 [of which 29 received CLES and 284 received DBS]; Fig. 1).

Of the 608 people meeting study eligibility, 53.3% were male, 93.1% were White, and 78.9% were aged 60 years or older (Table 1). More people who continued oral therapy were aged 70–79 years than were people who transitioned to device-aided therapy (38.6% vs. 28.1%, respectively), which was reflected in the difference in median age between each cohort (68.0 years [range, 48.0–93.0] and 66.0 years [range, 41.0–85.0], respectively; Table 1). The median time since PD diagnosis was 8.0 years (range, 0.0–34.0) among people who continued oral therapy and 10.0 years (range, 2.0–28.0) among people who transitioned to device-aided therapy (Table 1).

**Table 1:** Baseline demographic and clinical characteristics^a^

Characteristic	Continued oral therapy *n* = 295	Transitioned to device-aided therapy *n* = 313	Total *N* = 608
Sex, *N* (%)			
Male	157 (53.2)	167 (53.4)	324 (53.3)
Female	138 (46.8)	146 (46.6)	284 (46.7)
Ethnicity, *N* (%)			
Asian	3 (1.0)	4 (1.3)	7 (1.2)
Black	3 (1.0)	1 (0.3)	4 (0.7)
White	270 (91.5)	296 (94.6)	566 (93.1)
Hispanic	7 (2.4)	11 (3.5)	18 (3.0)
Other	12 (4.1)	1 (0.3)	13 (2.1)
Age, y, *N* (%)			
40–49	1 (0.3)	8 (2.6)	9 (1.5)
50–59	61 (20.7)	58 (18.5)	119 (19.6)
60–69	94 (31.9)	155 (49.5)	249 (41.0)
70–79	114 (38.6)	88 (28.1)	202 (33.2)
≥80	25 (8.5)	4 (1.3)	29 (4.8)
Median (range)	68.0 (48.0–93.0)	66.0 (41.0–85.0)	67.0 (41.0–93.0)
Time^b^ since PD diagnosis, y, median (range)	8.0 (0.0–34.0)	10.0 (2.0–28.0)	9.0 (0.0–34.0)
LEDD, mg, mean (SD)	1519.9 (458.5)	1485.1 (400.1)	1502.0 (429.4)

*LEDD* levodopa equivalent daily dose, *PD* Parkinson’s disease, *SD* standard deviation.

^a^Baseline was defined as the time at which people met the eligibility criteria (continued oral therapy) or the visit approximately 3 months before transitioning to device-aided therapy (transitioned to device-aided therapy).

^b^People may have had PD symptoms for several years before diagnosis at a movement disorder center.

### Device-aided therapy and QoL

**Fig. 2:**
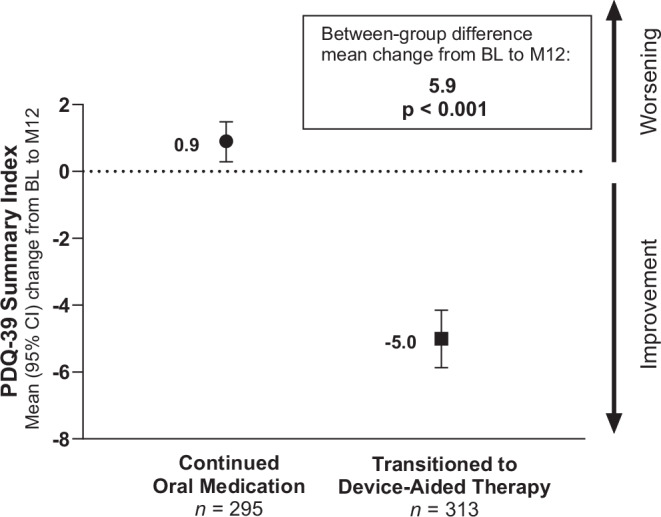
Mean change from baseline to month 12 in PDQ-39 summary index scores. BL baseline, CI confidence interval, M12 month 12, PDQ-39 39-item Parkinson’s Disease Questionnaire.

**Fig. 3:**
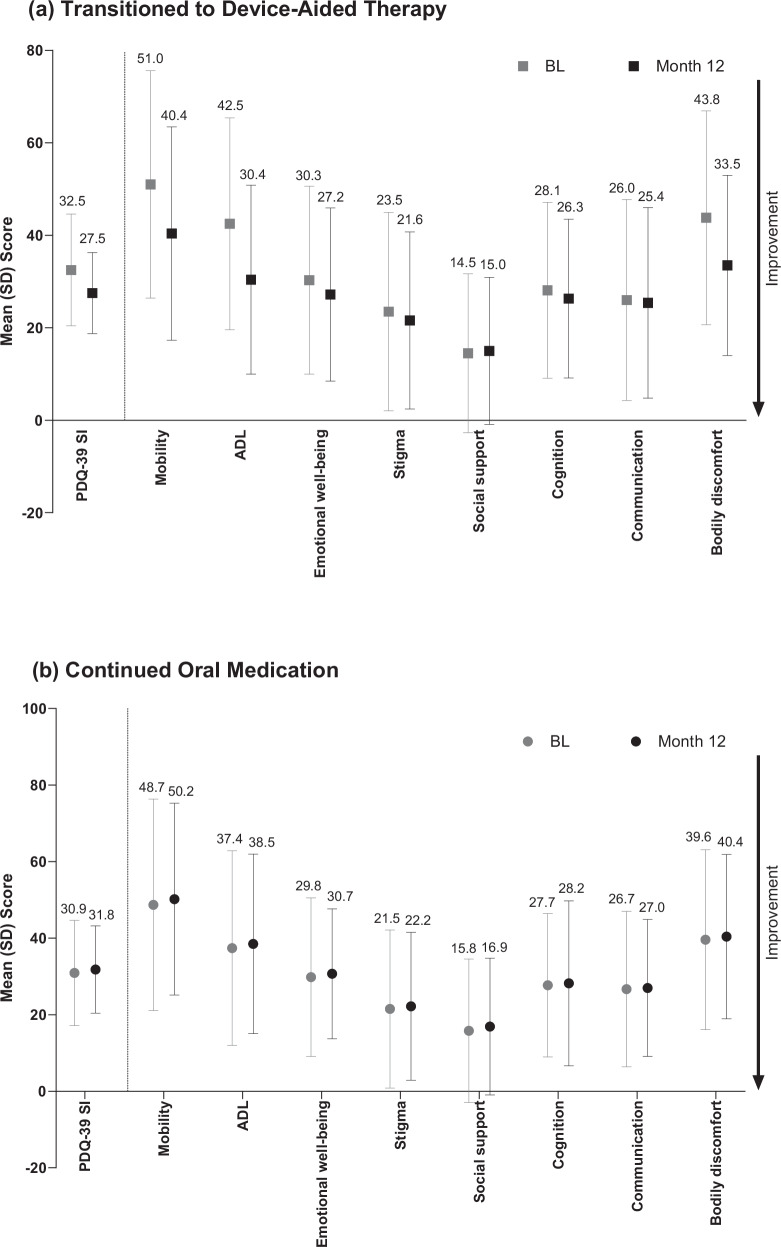
Mean change from baseline to month 12 in PDQ-39 domain scores. **a** People who transitioned to device-aided therapy. **b** People who continued oral therapy. ADL activities of daily living, BL baseline, PDQ-39 39-item Parkinson’s Disease Questionnaire, SD standard deviation, SI summary index.

At 12 months, people who transitioned to device-aided therapy demonstrated clinically meaningful^18^ improvements from baseline in QoL, as exhibited by a decrease in the 39-item Parkinson’s Disease Questionnaire (PDQ-39) summary index (SI) score (mean change from baseline, –5.0 [95% confidence interval [95% CI], –5.9 to –4.2]). In contrast, people who continued oral therapy showed a small increase in PDQ-39 SI scores (indicating worsening of QoL) at 12 months (0.9 [95% CI, 0.3–1.5]). Mean change from baseline in PDQ-39 SI scores differed significantly between cohorts (*p* < 0.001; Fig. 2). Scores in all PDQ-39 domains (except for social support) improved from baseline to 12 months for people who transitioned to device-aided therapy. The greatest improvements in QoL were observed in the domains of mobility, activities of daily living, and bodily discomfort (Fig. 3a). People who continued oral therapy had a slight worsening in QoL from baseline to 12 months across all PDQ-39 domain scores (Fig. 3b).

In sensitivity analyses, mean change from baseline to month 12 in PDQ-39 SI scores in the propensity score–matched population was –5.0 (95% CI, –5.9 to –4.1) among people who transitioned to device-aided therapy compared with 0.8 (95% CI, 0.2–1.5) among people who continued oral medication (*p* < 0.001). These findings are consistent with those observed in the unmatched population.

In subgroups of people aged <70 years and ≥70 years, improvements from baseline in QoL were also significantly greater in people who transitioned to device-aided therapy vs people who continued oral therapy (*p* < 0.0001 vs continued oral therapy for both subgroups; Supplementary Fig. 1). Changes from baseline in PDQ-39 domain scores within each age subgroup (< 70 years and ≥70 years) were also consistent with results observed in the overall population (Supplementary Fig. 2).

### Device-aided therapy and the longitudinal course of advanced PD

At baseline, UPDRS scores were similar across cohorts (Table 2), suggesting that people were not excluded from receiving device-aided therapy based on PD disease severity before surgery. Furthermore, mean scores for individual UPDRS items potentially related to contraindications for device-aided therapies (items 1, 2, 3, 5, 13, 18, 29, 30) were comparable between people who continued oral therapy and those who transitioned to device-aided therapy (Supplementary Table 1). A *t* test comparing UPDRS Part I total scores between the 2 groups yielded a *p* value of 0.4022, suggesting that total scores on Part I of the UPDRS were not correlated to selecting a specific therapy (continuing oral therapy or transitioning to device-aided therapy; Supplementary Table 2).

**Table 2:** Mean change from baseline to month 12 in UPDRS scores

UPDRS score	Continued oral therapy *n* = 295	Transitioned to device-aided therapy *n* = 313	*p* value^a^
UPDRS I score			
No.	195	233	
Baseline score	2.7	2.9	
Change from baseline, mean (95% CI)	0.2 (0.03 to 0.28)	0.1 (–0.09 to 0.19)	0.2914
UPDRS II score			
No.	249	257	
Baseline score	16.3	16.7	
Change from baseline, mean (95% CI)	1.6 (0.96 to 2.22)	–1.4 (–1.87 to –0.85)	< 0.001
UPDRS III total score			
No.	295	313	
Baseline score	42.7	42.6	
Change from baseline, mean (95% CI)	1.4 (–0.14 to 2.99)	–10.1 (–10.49 to –9.74)	< 0.001
UPDRS III gait sum score			
No.	295	313	
Baseline score	5.8	5.8	
Change from baseline, mean (95% CI)	1.1 (0.84 to 1.44)	0.2 (0.17 to 0.31)	< 0.001
UPDRS III bradykinesia sum score			
No.	295	313	
Baseline score	15.8	15.4	
Change from baseline, mean (95% CI)	–0.0 (–0.70 to 0.67)	–4.7 (–4.89 to –4.42)	< 0.001
UPDRS IV dyskinesia sum score (items 32–34)			
No.	295	313	
Baseline score	2.6	2.6	
Change from baseline, mean (95% CI)	0.1 (0.01 to 0.15)	–1.6 (–1.84 to –1.44)	< 0.001
UPDRS IV total score (items 32–34, 39)			
No.	295	313	
Baseline score	4.1	4.0	
Change from baseline, mean (95% CI)	0.1 (0.00 to 0.21)	–2.2 (–2.41 to –1.92)	< 0.001
UPDRS IV, item 39			
No.	295	313	
Baseline			
0 (none)	0 (0.0)	0 (0.0)	
1 (1%–25% of day)	179 (60.7)	204 (65.2)	
2 (26%–50% of day)	79 (26.8)	70 (22.4)	
3 (51%–75% of day)	37 (12.5)	39 (12.5)	
4 ( ≥ 76% of day)	0 (0.0)	0 (0.0)	
Change from baseline, *N* (%)			
Improved (decreased score)	23 (7.8)	145 (46.3)	< 0.001
No change	228 (77.3)	145 (46.3)	
Worsened (increased score)	44 (14.9)	23 (7.8)	
UPDRS total score (without items 35–38, 40–42)			
No.	195	233	
Baseline score	64.4	66.2	
Change from baseline, mean (95% CI)	3.0 (0.76 to 5.15)	–13.4 (–14.12 to –12.58)	< 0.001

UPDRS Part I measures mentation, behavior, and mood (score range, 0–16); Part II measures activities of daily living (score range, 0–52); Part III measures motor examination (score range, 0–108); and Part IV measures complications of therapy (score range, 0–23). Higher scores in all UDPRS domains indicate more impairment or complications. The UPDRS total score ranges from 0 (absence of signs and symptoms of PD) to 199 (most severe level of disability due to PD).

*CI* confidence interval, *UPDRS* Unified Parkinson’s Disease Rating Scale.

^a^Comparison between cohorts, *t* test with α = 0.05 (except UPDRS IV, item 39, which used χ^2^ test).

After 12 months, there was no difference between cohorts in UPDRS Part I scores (cognitive and emotional domains). However, UPDRS Part II (activities of daily living), Part III (motor scores), and Part IV (treatment complications) scores were significantly improved (all *p* < 0.001) from baseline among people who transitioned to device-aided therapy vs. people who continued oral therapy, in which all UPDRS scores remained unchanged or showed some worsening (Table 2). While UPDRS Part III gait sum score slightly worsened in both groups, there was a significant difference between the cohorts, with greater worsening noted among people who continued oral therapy than among people who transitioned to device-aided therapy.

For UPDRS Part IV, item 39 (“What proportion of the waking day is the patient ‘Off’ on average?”) at baseline, there were no people with a value of 0 (none), and there were slightly more people who transitioned to device-aided therapy than who continued oral medication with a value of 1 (1%–25% of day; 65.2% vs. 60.7%, respectively; Table 2). At month 12, more people who transitioned to device-aided therapy than continued oral medication had a value of 0 (24.3% vs. 0.3%, respectively). Significantly greater proportions of people who transitioned to device-aided therapy (46.3%) than continued oral medication (7.8%) showed 12-month improvements in UPDRS Part IV item 39 scores (*p* < 0.001; Table 2). People who transitioned to device-aided therapy had significantly better improvement in dyskinesia (UPDRS Part IV items 32–34) at 12 months than those who continued oral medication (dyskinesia sum score, mean [95% CI], –1.6 [–1.84 to –1.44] vs 0.1 [0.01–0.15], respectively; *p* < 0.001; Table 2).

Consistent with results from the overall population, improvements from baseline in UPDRS Parts II–IV scores were significantly greater in people who transitioned to device-aided therapy vs those who continued oral medication in both age subgroups (*p* < 0.01 for both people aged <70 years and ≥70 years; Supplementary Table 3).

### Device-aided therapy and medication usage

From baseline to 12 months, people who transitioned to device-aided therapy showed a reduction in the median number of oral PD medications, median levodopa equivalent daily dose (LEDD) per person, and proportion of people using any rescue medications. People who continued oral medication showed increased LEDD, with similar numbers of oral medications and rates of rescue medication use (Supplementary Table 4). The median LEDD decreased from 1445.8 mg (baseline) to 1200.0 mg (month 12) in people who transitioned to DBS and decreased from 1790.4 mg (baseline) to 1000.0 mg (month 12) in people who transitioned to CLES.

### Device-aided therapy and activities of daily living

The Schwab and England Activities of Daily Living Index was used for only a few people (continued oral medication, *n* = 24; transitioned to device-aided therapy, *n* = 22). Nevertheless, despite the small number of people in each cohort, there was a skew toward more independence at 12 months among people who transitioned to device-aided therapy (Supplementary Table 5). All people who transitioned to device-aided therapy showed improvement in independence on the Schwab and England scale at 12 months, compared with only one patient who continued oral medication (*p* < 0.001; Supplementary Table 5).

## Discussion

To our knowledge, this is the largest single-center study assessing QoL in people with PD after transitioning to device-aided therapy. Findings from the present study add to the existing evidence showing the potential clinical benefit for people living with PD with motor fluctuations who transitioned to device-aided therapies^15^. In this retrospective, large cohort analysis, people with advanced PD accompanied by motor fluctuations who transitioned to device-aided therapy showed marked improvement in QoL and motor symptoms at 12 months, regardless of demographic or disease characteristics, while matched people (matched based on clinical characteristics, demographics, device-aided therapy eligibility, and study inclusion/exclusion criteria) who remained on oral treatment did not improve or slightly worsened as a cohort. The improvements in QoL observed in people who transitioned to device-aided therapy exceeded the minimal clinically important improvement in PDQ-39 of 4.7 points^18^. The improvement in QoL manifested in our cohort was consistent with the findings from a network meta-analysis of best medical care and device-aided therapies, which found that improvements in PDQ-39 SI scores from baseline to 6 months were greater in all the groups receiving device-aided therapy when compared with best medical treatment^11^. These findings are also consistent with randomized controlled trials of DBS or CLES when compared with best medical therapy^19–21^.

The singular PDQ-39 domain which failed to improve was the social support domain. The lack of improvement was possibly due to many factors including the need for more support with device-aided therapies. Notably, the use of CLES and DBS have both been shown to reduce long-term burden for care partners^22,23^.

People with PD who transitioned to device-aided therapy showed significant improvements in UPDRS Parts II-IV (activities of daily living, motor symptoms [eg, bradykinesia], and complications of therapy) scores compared with those who remained on oral therapy. These findings are consistent with those from other studies comparing device-aided therapy with oral medication, which similarly demonstrated significant improvement in UPDRS Part II scores and nominal improvement in UPDRS Part III scores among people receiving CLES, and significant improvements in UPDRS Part III and Part IV scores among people receiving DBS^13,20,21^. While the literature is less clear regarding the effects of DBS vs. oral medication on UPDRS Part II scores, there have been improvements when compared to pre-surgical data^24^. Collectively, these results affirm the benefits of device-aided therapies on activities of daily living and motor symptoms. There was no worsening in cognition or psychosis at 1 year (UPDRS Part I scores) between groups, though our cohort did not undergo formal neuropsychological testing, and it is possible that selection bias for surgery played a role.

While our cohort matched individuals eligible for surgery with age-matched controls who, in general, had low concerns for prominent cognitive issues, it is possible that the oral therapy group included people with relative contraindications that made device-aided therapies less advisable—symptoms that are often associated with worsened quality of life. To address this potential selection bias, we compared baseline scores for UPDRS items related to potential factors precluding patients from device-aided therapy consideration and conducted a correlation analysis to determine whether UPDRS Part I total scores were associated with therapy choice. We found that individual UPDRS item scores were comparable across the 2 cohorts, even in people aged ≥70 years, and that there was no correlation between UPDRS Part I total scores and therapy choice. These findings suggest that people were evenly matched between the 2 cohorts and that selection bias was minimal.

Despite the established benefits of device-aided therapy, many potentially eligible people may remain on complex oral regimens^17^. Many factors contribute to the current lack of implementation, including access to specialized centers and education about potential benefits. Another barrier may be the lack of commonly accepted criteria or guidelines to define optimal candidates^25^. Although there is evidence that some people may benefit from earlier initiation of device-aided therapies^26,27^, clinicians may find it difficult to assess a person’s disease stage because of a lack of time or resources^28^. Finally, as people with PD age and comorbidities increase, they may miss the window for greater benefit with device-aided therapies^28^. While this study had a higher proportion of people aged 70–79 years in the oral therapy group compared with those who transitioned to device-aided therapy, subgroup analyses indicated that findings were similar in people aged <70 years and ≥70 years and were consistent with those of the overall population.

Screening tools have been developed to aid in timely identification of people who may benefit from device-aided therapies; however, these tools are not diagnostic and are intended to complement physician judgment^28,29^. Clear guidelines may help in the future with identifying people who might benefit from device-aided therapy^28^.

Although the reasons driving the decision to continue oral treatment or transition to device-aided therapy were not directly examined in the present study, potential factors guiding the decision have been previously explored^30^. Reasons include differences in access and availability in different countries and regions, a person’s fear of surgery, a delayed referral to specialists that utilize device-aided therapies, physician biases related to familiarity with device-aided therapy or diagnostic criteria for transitioning from oral treatment, people’s preferences, and lack of information^28,31,32^. Finally, it is possible that with the development of subcutaneous infusion therapies, including continuous subcutaneous administration of levodopa/carbidopa and apomorphine, these less invasive alternatives may aid in easier initiation and implementation of device-aided therapies^33–35^. Finally, some clinicians have suggested that earlier shared decision-making will increase awareness so that when a transition is needed, it can be rapid and smooth^28^.

Limitations of this study include the retrospective analysis of clinical data and patient-reported outcomes, which are impacted by the quality of available data being highly dependent on the accuracy of data entry personnel. Potential selection bias, missing data, and the extent to which the data accurately reflect the broader population further contribute to the limitations. The reasons driving the selection of the therapy were not directly captured. Candidacy and counseling for advanced therapies in PD are inherently complex due to the heterogeneous nature of the disease, variability in patient goals, medication tolerability, and social and financial circumstances. As such, treatment decisions must be highly individualized. Our multidisciplinary team brings over 2 decades of experience in guiding patients through these decisions, but further research is warranted to explore the attitudes and considerations surrounding the decisions to initiate device-aided therapies or continue oral treatment. Additionally, there was no systematic collection of safety data; thus, no benefit-risk analysis can be performed for the 2 treatment cohorts. The treating team managed side effects in accordance with the clinical standard of care. Observed adverse events were consistent with those commonly reported in the literature, and no unexpected events were encountered. Both CLES and DBS have been known to elicit device- and surgery-related safety risks, which should be considered when making treatment decisions^36^. Data from our study were retrieved from a single center that was highly specialized in the treatment of neurologic diseases. Also, to receive a specific device-aided therapy, people must have gone through a complete multidisciplinary screening, which likely eliminated the cohort of people with low responsiveness to levodopa and significant cognitive dysfunction. Finally, matching control groups to candidates for device-aided therapy has proved challenging as many who remain in the oral medication group may be too early or too late for appropriate transitioning to a more invasive approach.

In this retrospective, observational study conducted in a real-world setting, people with advanced PD who transitioned to device-aided therapy demonstrated significant, clinically meaningful improvements in QoL. Additionally, the cohort transitioning to device-aided therapy also showed improvements in activities of daily living and motor fluctuations when compared with those remaining on oral therapy. Although this trial was not randomized and matching to the control group was challenging, clinicians should consider that people with PD who experience bothersome motor fluctuations and dyskinesia despite optimization of their oral therapy may be a candidate for device-aided treatment.

## Methods

### Study design

This was a retrospective, secondary cohort study that reviewed medical records from people with PD from the University of Florida Norman Fixel Institute for Neurological Disease INFORM database (Gainesville, United States). The INFORM database was approved by the University of Florida institutional review board and is a clinical registry of prospectively collected data from people with movement disorders. People who saw specialists at the clinic had the option to allow their data to be included in the central database and provided informed consent to participate. All included data from people with PD were collected at the clinic during routine visits from 2012 to 2021. This time frame was chosen to account for the increased experience the clinic accrued in working with DBS, implementation of flowsheets in medical records, and to incorporate a higher proportion of people receiving CLES, which was approved in 2015.

### People and treatment

Eligible people were adults aged 40–80 years experiencing idiopathic PD for >5 years, with diagnosis confirmed by a movement disorder specialist. To determine people with advanced PD who were eligible for device-aided therapy, proxies close to criteria published in the literature^30^ and clinician personal practice/judgment were utilized. In the absence of “On” and “Off” time data in the registry, eligible people with PD had to have moderate motor fluctuations and dyskinesia, as defined by the UPDRS Part IV, items 32 (“What proportion of the waking day are dyskinesias present?”) and 39 (“What proportion of the waking day is the person ‘Off’ on average?”)^37^, present for ≥25% of the day.

People with PD were currently receiving treatment with carbidopa/levodopa four times/day or more (or extended-release carbidopa/levodopa [Rytary, Amneal Pharmaceuticals LLC, Bridgewater, NJ] three times/day or more) or cumulative levodopa equivalent dose >400 mg/day. People were allocated to one of two cohorts based on the treatment received: those who received continuous oral dopaminergic therapy and those who transitioned to device-aided therapy (DBS or CLES) for management of their symptoms. Cohorts were matched based on clinical characteristics, demographics, device-aided therapy eligibility, and study inclusion/exclusion criteria.

People with PD were required to have continuous documentation in the registry for ≥12 months before data capture, with people who transitioned to device-aided therapy also required to have had one or more visits approximately 3 months prior to initiation of device-aided therapy.

Exclusion criteria included people with a diagnosis of atypical Parkinsonism, confirmed by a movement disorder specialist, or those participating in an interventional trial.

### Assessments

The primary end point was the mean change from baseline to month 12 in the patient-reported, PDQ-39^38^ SI score (calculated as the sum of dimension total scores divided by eight). Secondary end points included changes from baseline to month 12 in the physician-administered UPDRS Parts I–IV^37^, the physician-administered Schwab and England Activities of Daily Living Index^39^, the number of oral PD medications used, the LEDD, and the use of rescue medications. To determine if there were differences in results by age, the mean changes from baseline to month 12 in PDQ-39 SI scores, PDQ-39 domain scores, and UPDRS Parts I–IV were assessed post hoc in subgroups of people aged <70 years and ≥70 years. A post hoc correlation analysis was conducted to assess whether baseline UPDRS Part I scores were associated with the decision to continue oral therapy vs transition to device-aided therapy.

### Analyses

The full analysis set comprised all enrolled people who fulfilled the eligibility criteria and had an evaluation of PDQ-39 at baseline and at month 12 from the INFORM database. Baseline was defined as the visit 3 months before initiating device-aided therapy (for people who transitioned) or within 6 months of the time at which people met the eligibility criteria (for people who continued oral therapy). The baseline for the device-aided therapy cohort was defined this way to capture the patient’s clinical status at the time they ultimately decided to pursue the intervention.

The sample size for this exploratory analysis was based on the number of people in the database who both met eligibility requirements and had complete PDQ-39 measurements at baseline and month 12. A sample size of 350 people who transitioned to device-aided therapy and 314 matched controls would provide 89.5% power to detect a difference between cohorts for the primary end point based on an assumed mean (standard deviation [SD]) between-cohort difference in PDQ-39 score of 3 (12) with a 5% two-sided level of significance.

Continuous variables (PDQ-39 and UPDRS) were summarized using descriptive statistics (mean and SD; median and range). Ordinal categorical variables (Schwab and England Activities of Daily Living Index) were presented by frequency distributions. Percentages were calculated based on the number of nonmissing observations. Two-sided 95% CIs for the estimated means were provided for primary and secondary end points. The 95% CI was also calculated for each difference in mean between cohorts. Statistical comparison between cohorts was performed using *t* tests for continuous data and the χ^2^ test for categorical data. Alpha was set to 5%. Statistical analyses were conducted using SAS statistical software, version 9.4 or higher (SAS Institute, Cary, NC, USA).

Sensitivity analyses were performed for the primary end point, applying propensity score matching. Propensity scores were estimated using a multiple logistic regression model for all people enrolled. People in both cohorts were propensity score–matched based on selected clinical and demographic characteristics (sex, age, time since PD diagnosis, presence of moderate motor fluctuations and dyskinesia ≥ 25% of the day per UDPRS IV items 32 and 39, and current use of levodopa four or more times/day [or carbidopa/levodopa three or more times/day], or cumulative levodopa equivalent dose >400 mg/day). The SAS procedure PSMATCH with a caliper level of 0.2 was used.

## Supplementary information

Supplemental Material

## Data Availability

AbbVie is committed to responsible data sharing regarding the studies we sponsor. This includes access to anonymized individual and trial-level data (analysis data sets), as well as other information (eg, protocols, clinical study reports, or analysis plans), as long as the trials are not part of an ongoing or planned regulatory submission. This includes requests for clinical trial data for unlicensed products and indications. These registry data can be requested by any qualified researchers who engage in rigorous, independent, scientific research, and will be provided following review and approval of a research proposal and Statistical Analysis Plan (SAP) and execution of a Data Sharing Agreement (DSA). Data requests can be submitted at any time after approval in the United States and Europe and after acceptance of this manuscript for publication. The data will be accessible for 12 months, with possible extensions considered. For more information on the process or to submit a request, visit the following link: https://vivli.org/ourmember/abbvie/, then select “Home”.
