# Supplementary material for: Parkinson’s disease quality of life at 12 months comparing invasive device-aided therapy with oral treatment
Source: NPJ Parkinsons Dis. 2025 Aug 11;11:235. doi: 10.1038/s41531-025-01093-x (PMC12339671; doi:10.1038/s41531-025-01093-x)

**SUPPLEMENTAL MATERIAL**

**Parkinson’s disease quality of life at 12 months comparing invasive device-aided therapy with oral treatment**

Running title: Quality of life of device-aided therapies in Parkinson’s

Adolfo Ramirez-Zamora^1🖂^, Michael S. Okun^1^, Pavnit Kukreja^2^ and Wei Hu^1^

^1^Department of Neurology, Norman Fixel Institute for Neurological Diseases, University of Florida, Gainesville, FL, USA. ^2^AbbVie Inc., North Chicago, IL, USA.

**Corresponding Author:** Adolfo Ramirez-Zamora, MD, Norman Fixel Institute for Neurological Diseases, University of Florida, 3009 SW Williston Rd, Gainesville, FL 32608 ([Adolfo.Ramirez-Zamora@neurology.ufl.edu](mailto:Adolfo.Ramirez-Zamora@neurology.ufl.edu)); Phone: +1-352-294-5400

| **Supplemental Table 1.** Select UPDRS item scores at baseline by age | | | | |
| --- | --- | --- | --- | --- |
|  | Continued Oral Therapy | | Transitioned to Device-Aided Therapy | |
| UPDRS item score, mean (SD) | Age <70 years  (*n* = 108) | Age ≥70 years  (*n* = 87) | Age <70 years (*n* = 173) | Age ≥70 years (*n* = 60) |
| Item 1 | 0.8 (0.66) | 1.0 (0.93) | 0.8 (0.61) | 1.0 (0.58) |
| Item 2 | 0.5 (0.77) | 0.7 (0.76) | 0.5 (0.73) | 0.8 (0.77) |
| Item 3 | 0.5 (0.56) | 0.5 (0.63) | 0.7 (0.63) | 0.6 (0.61) |
| Item 5 | 0.7 (0.81)^a^ | 0.8 (0.87)^b^ | 1.1 (0.85)^c^ | 1.0 (0.75)^d^ |
| Item 13 | 1.1 (0.99)^e^ | 1.1 (0.99)^f^ | 1.0 (0.90)^c^ | 1.1 (0.78)^d^ |
| Item 15 | 1.7 (1.04)^e^ | 1.8 (1.05)^f^ | 1.8 (1.02)^c^ | 1.9 (1.00)^d^ |
| Item 18 | 0.8 (1.07)^g^ | 1.0 (1.11)^e^ | 1.1 (1.11)^h^ | 1.1 (1.03)^i^ |
| Item 29 | 2.2 (0.49)^g^ | 2.2 (0.51)^e^ | 2.1 (0.45)^h^ | 2.2 (0.47)^i^ |
| Item 30 | 2.0 (0.66)^g^ | 1.9 (0.67)^e^ | 2.0 (0.60)^h^ | 2.0 (0.61)^i^ |
| *UPDRS* Unified Parkinson’s Disease Rating Scale.  ^a^*n* = 138. ^b^*n* = 109. ^c^*n* = 190. ^d^*n* = 67. ^e^*n* = 139. ^f^*n* = 110. ^g^*n* = 156. ^h^*n* = 139. ^i^*n* = 92. | | | | |

| **Supplemental Table 2.** Correlation analysis of UPDRS I total score at baseline vs therapy choice | | | |  |
| --- | --- | --- | --- | --- |
| Variable | Continued Oral Therapy  (*n* = 195) | Transitioned to Device-Aided Therapy  (*n* = 233) | *p* value^a^ | |
| UPDRS I total score, n (%) |  |  |  | |
| 0 | 20 (10.3) | 23 (9.9) |  | |
| 1 | 31 (15.9) | 33 (14.2) |  | |
| 2 | 44 (22.6) | 41 (17.6) |  | |
| 3 | 45 (23.1) | 47 (20.2) |  | |
| 4 | 27 (13.8) | 56 (24.0) |  | |
| 5 | 12 (6.2) | 18 (7.7) |  | |
| 6 | 9 (4.6) | 8 (3.4) |  | |
| 7 | 4 (2.1) | 5 (2.1) |  | |
| 8 | 1 (0.5) | 1 (0.4) |  | |
| 9 | 1 (0.5) | 1 (0.4) |  | |
| 10 | 0 | 0 |  | |
| 11 | 1 (0.5) | 0 (0.0) |  | |
| 12 | 0 | 0 |  | |
| 13 | 0 | 0 |  | |
| 14 | 0 | 0 |  | |
| 15 | 0 | 0 |  | |
| 16 | 0 | 0 |  | |
| UPDRS I total score, mean (SD) | 2.7 (1.9) | 2.9 (1.8) | 0.4022 | |
| *UPDRS* Unified Parkinson’s Disease Rating Scale.  ^a^Comparison between cohorts (*t* test). | | | |  |

| **Supplemental Table 3.** Mean change from baseline to month 12 in UPDRS scores by age | | | | | | |  |  |  |
| --- | --- | --- | --- | --- | --- | --- | --- | --- | --- |
| UPDRS Score | Age <70 years | | | Age ≥70 years | | | |  |  |
|  | Continued Oral Therapy | Transitioned to Device-Aided Therapy | *p* value^a^ | Continued Oral Therapy | Transitioned to Device-Aided Therapy | *p* value^a^ | | |  |
| UPDRS I score |  |  |  |  |  |  | | |  |
| No. | 108 | 173 |  | 87 | 60 |  | | |  |
| Baseline score | 2.6 | 2.8 |  | 2.9 | 3.0 |  | | |  |
| Change from baseline, mean (95% CI) | 0.2 (0.04 to 0.35) | 0.1 (−0.08 to 0.27) | 0.2930 | 0.1 (−0.11 to 0.32) | −0.1 (−0.30 to 0.17) | 0.3881 | | |  |
| UPDRS II score |  |  |  |  |  |  | | |  |
| No. | 139 | 190 |  | 110 | 67 |  | | |  |
| Baseline score | 16.0 | 16.5 |  | 16.7 | 17.2 |  | | |  |
| Change from baseline, mean (95% CI) | 1.4 (0.57 to 2.21) | −1.2 (−1.77 to −0.58) | < 0.0001 | 1.8 (0.84 to 2.85) | −1.9 (−2.89 to −0.87) | < 0.0001 | | |  |
| UPDRS III total score |  |  |  |  |  |  | | |  |
| No. | 156 | 221 |  | 139 | 92 |  | | |  |
| Baseline score | 42.8 | 42.6 |  | 42.5 | 42.7 |  | | |  |
| Change from baseline, mean (95% CI) | 1.2 (−1.06 to 3.47) | −10.1 (−10.60 to −9.68) | < 0.0001 | 1.7 (−0.49 to 3.84) | −10.1 (−10.72 to −9.41) | < 0.0001 | | |  |
| UPDRS III gait sum score |  |  |  |  |  |  | | |  |
| No. | 156 | 221 |  | 139 | 92 |  | | |  |
| Baseline score | 5.8 | 5.7 |  | 5.7 | 5.9 |  | | |  |
| Change from baseline, mean (95% CI) | 1.0 (0.54 to 1.39) | 0.3 (0.18 to 0.33) | < 0.0001 | 1.3 (0.91 to 1.77) | 0.2 (0.04 to 0.35) | < 0.01 | | |  |
| UPDRS III bradykinesia sum score |  |  |  |  |  |  | | |  |
| No. | 156 | 221 |  | 139 | 92 |  | | |  |
| Baseline score | 15.8 | 15.4 |  | 15.7 | 15.5 |  | | |  |
| Change from baseline, mean (95% CI) | −0.1 (−1.16 to 0.86) | −4.5 (−4.83 to −4.27) | < 0.0001 | 0.1 (−0.80 to 1.05) | −4.9 (−5.34 to −4.46) | < 0.0001 | | |  |
| UPDRS IV dyskinesia sum score (items 32–34) |  |  |  |  |  |  | | |  |
| No. | 156 | 221 |  | 139 | 92 |  | | |  |
| Baseline score | 2.6 | 2.6 |  | 2.5 | 2.6 |  | | |  |
| Change from baseline, mean (95% CI) | 0.1 (0.01 to 0.21) | −1.7 (−1.93 to −1.44) | < 0.0001 | 0 (−0.06 to 0.15) | −1.5 (−1.87 to −1.17) | < 0.0001 | | |  |
| UPDRS IV total score  (items 32–34, 39) |  |  |  |  |  |  | | |  |
| No. | 156 | 221 |  | 139 | 92 |  | | |  |
| Baseline score | 4.2 | 4.1 |  | 4.0 | 3.9 |  | | |  |
| Change from baseline, mean (95% CI) | 0.1 (−0.03 to 0.24) | −2.3 (−2.55 to −1.96) | < 0.0001 | 0.1 (−0.05 to 0.27) | −1.9 (−2.36 to −1.53) | < 0.0001 | | |  |
| UPDRS IV, item 39 |  |  |  |  |  |  | | |  |
| No. | 156 | 221 |  | 139 | 92 |  | | |  |
| Baseline |  |  |  |  |  |  | | |  |
| 0 (none) | 0 | 0 |  | 0 | 0 |  | | |  |
| 1 (1%–25% of day) | 93 (59.6) | 136 (61.5) |  | 86 (61.9) | 68 (73.9) |  | | |  |
| 2 (26%–50% of day) | 46 (29.5) | 52 (23.5) |  | 33 (23.7) | 18 (19.6) |  | | |  |
| 3 (51%–75% of day) | 17 (10.9) | 33 (14.9) |  | 20 (14.4) | 6 (6.5) |  | | |  |
| 4 (≥ 76% of day) | 0 | 0 |  | 0 | 0 |  | | |  |
| Change from baseline, *N* (%) |  |  |  |  |  |  | | |  |
| Improved (decreased score) | 13 (8.3) | 105 (47.5) | < 0.0001 | 10 (7.2) | 40 (43.5) | < 0.0001 | | |  |
| No change | 124 (79.5) | 100 (45.2) |  | 104 (74.8) | 45 (48.9) |  | | |  |
| Worsened (increased score) | 19 (12.2) | 16 (7.2) |  | 25 (18.0) | 7 (7.6) |  | | |  |
| UPDRS total score  (without items 35–38, 40–42) |  |  |  |  |  |  | | |  |
| No. | 108 | 173 |  | 87 | 60 |  | | |  |
| Baseline score | 64.5 | 66.1 |  | 64.3 | 66.8 |  | | |  |
| Change from baseline, mean (95% CI) | 2.7 (−0.41 to 5.76) | −13.4 (−14.32 to −12.45) | < 0.0001 | 3.3 (0.15 to 6.47) | −13.3 (−14.59 to −11.94) | < 0.0001 | | |  |
| *CI* confidence interval, *UPDRS* Unified Parkinson's Disease Rating Scale.  UPDRS Part I measures mentation, behavior, and mood (score range, 0–16); Part II measures activities of daily living (score range, 0–52); Part III measures motor examination (score range, 0–108); and Part IV measures complications of therapy (score range, 0–23). Higher scores in all UDPRS domains indicate more impairment or complications. The UPDRS total score ranges from 0 (absence of signs and symptoms of PD) to 199 (most severe level of disability due to PD).  ^a^Comparison between cohorts, *t* test with α = 0.05 (except UPDRS IV, item 39, which used χ² test). | | | | | | | | | |

| **Supplemental Table 4.** Number of oral PD medications, LEDD, and use of rescue medications | | | | |
| --- | --- | --- | --- | --- |
|  | Continued Oral Therapy  *n* = 294 | | Transitioned to Device-Aided Therapy  *n* = 313 | |
| Oral PD Medications | Baseline | Month 12 | Baseline | Month 12 |
| Number of oral PD medications per person, median (range) | 3 (1–6) | 3 (1–6) | 3 (1–5) | 2 (1–5) |
| LEDD, mg, median (range) | 1425.2  (350.0–2980.4) | 1500.3  (350.0–3005.2) | 1450.4  (75.0–3130.0) | 1187.9  (25.0–2330.0) |
| DBS (*n* = 284) | — | — | 1445.8 (75.0–3130.0) | 1200.0 (25.0–2330.0) |
| CLES (*n* = 29) | — | — | 1790.4 (1070.4–2780.4) | 1000.0 (600.0–1450.0) |
| People taking any rescue medication, *N* (%) | 13 (4.4)^a^ | 13 (4.4)^a^ | 16 (5.1) | 10 (3.2) |
| *DBS* deep brain stimulation, *CLES* carbidopa-levodopa enteral suspension, *LEDD* levodopa equivalent daily dose, *PD* Parkinson’s disease.  ^a^*n* = 295 | | | | |

| **Supplemental Table 5.** Mean change from baseline to month 12 in Schwab and England Activities of Daily Living Scale | | | | | |
| --- | --- | --- | --- | --- | --- |
|  | Continued Oral Therapy, *N* (%)  *n* = 24 | | Transitioned to Device-Aided Therapy, *N* (%)  *n* = 22 | |  |
| Schwab and England Scale,  % of independence | Baseline | Month 12 | Baseline | Month 12 |  |
| 30 | 0 (0.0) | 0 (0.0) | 1 (4.5) | 0 (0.0) |  |
| 40 | 3 (12.5) | 4 (16.7) | 1 (4.5) | 0 (0.0) |  |
| 50 | 8 (33.3) | 9 (37.5) | 6 (27.3) | 1 (4.5) |  |
| 60 | 8 (33.3) | 9 (37.5) | 11 (50.0) | 3 (13.6) |  |
| 70 | 4 (16.7) | 2 (8.3) | 2 (9.1) | 6 (27.3) |  |
| 80 | 1 (4.2) | 0 (0.0) | 1 (4.5) | 9 (40.9) |  |
| 90 | 0 (0.0) | 0 (0.0) | 0 (0.0) | 3 (13.6) |  |
| Change from baseline | Continued Oral Therapy, *N* (%) | | Transitioned to Device-Aided Therapy, *N* (%) | | *p* value^a^ |
| Improved (increased score) | 1 (4.2) | | 22 (100.0) | | < 0.001 |
| No change | 17 (70.8) | | 0 (0.0) | |  |
| Worsened (decreased score) | 6 (25.0) | | 0 (0.0) | |  |
| ^a^Comparison between cohorts, χ² test with α = 0.05. | | | | | |

**Supplemental Figure 1.** Mean change from baseline to month 12 in PDQ-39 summary index scores by age
*BL* baseline, *CI* confidence interval, *M12* month 12, *PDQ-39* 39-item Parkinson’s Disease Questionnaire.


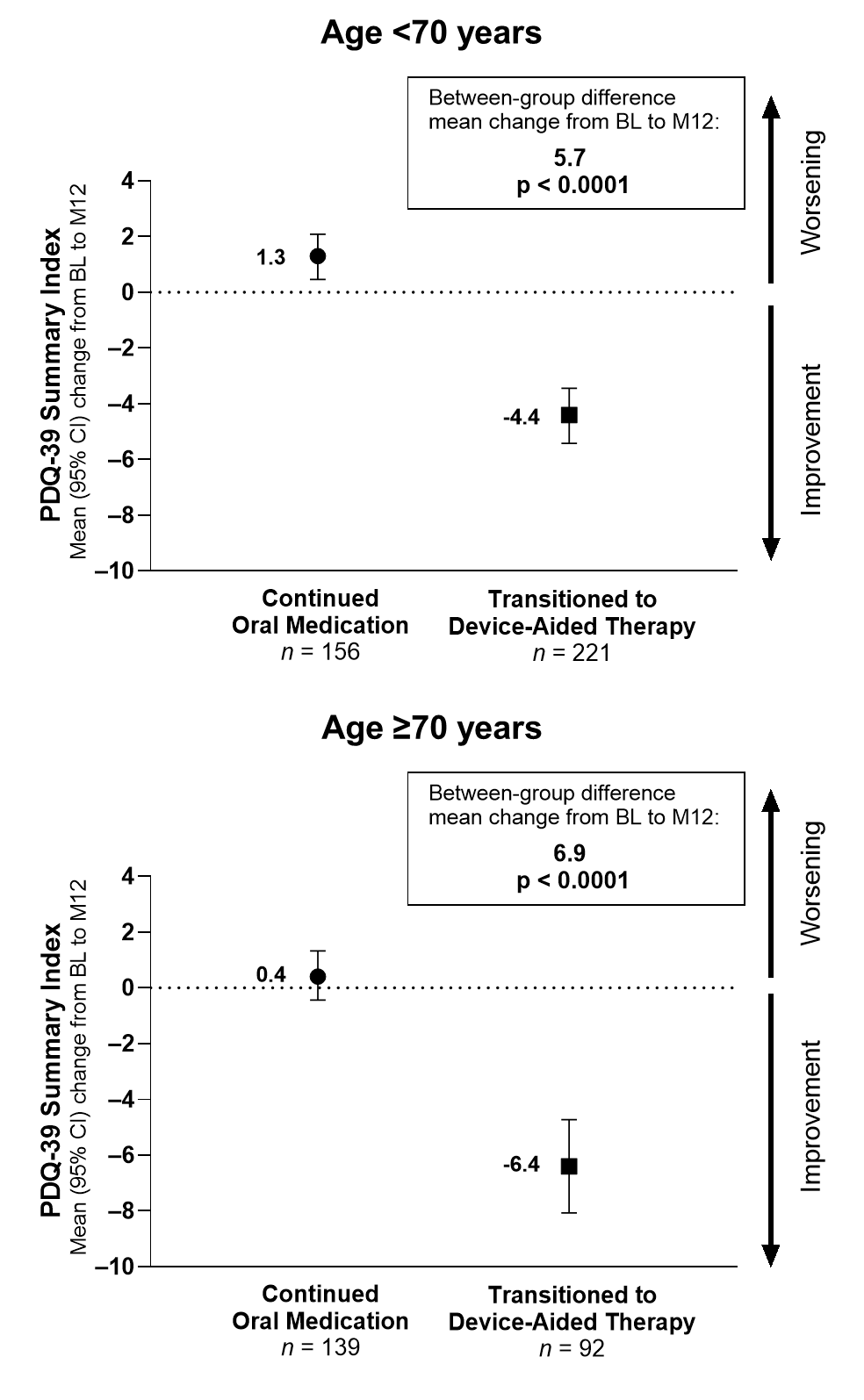


**Supplemental Figure 2. Mean change from baseline to month 12 in PDQ-39 domain scores by age.** *ADL* activities of daily living, *BL* baseline, *PDQ-39* 39-item Parkinson’s Disease Questionnaire, *SD* standard deviation, *SI* summary index.


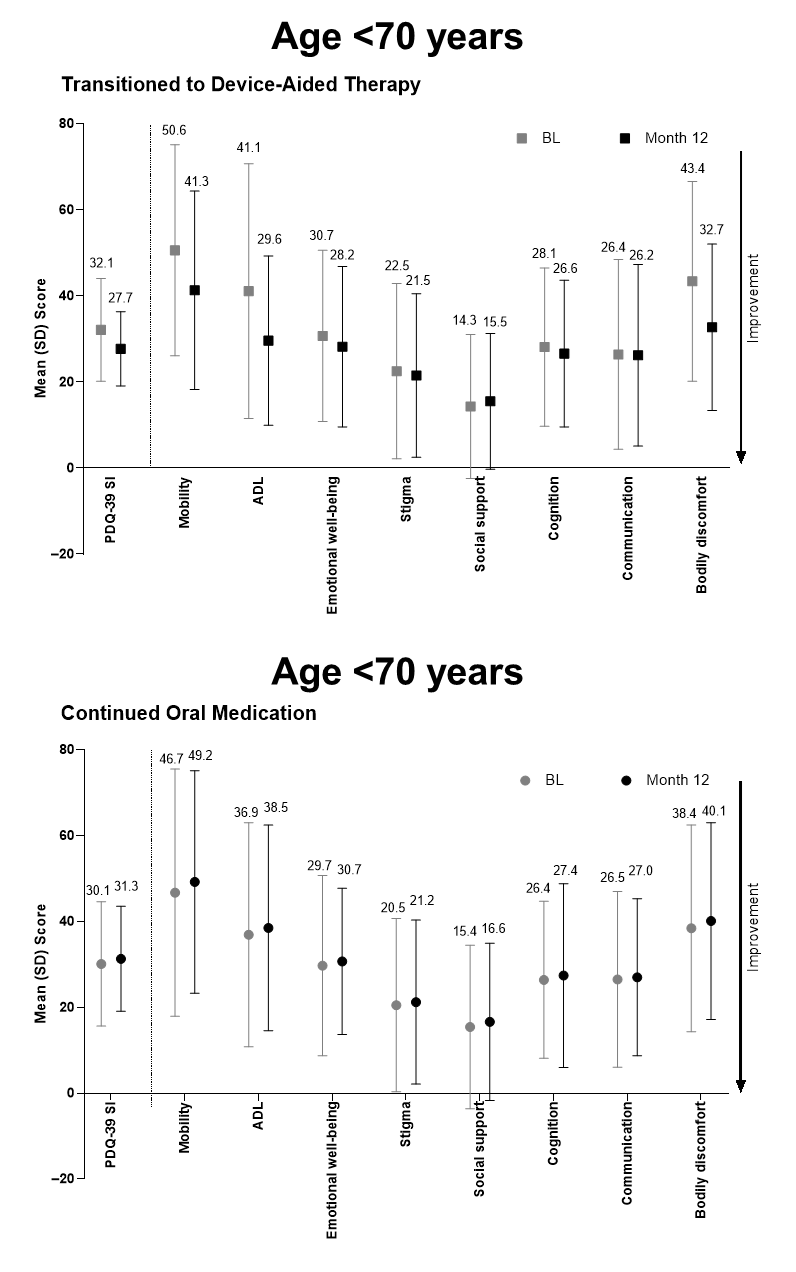

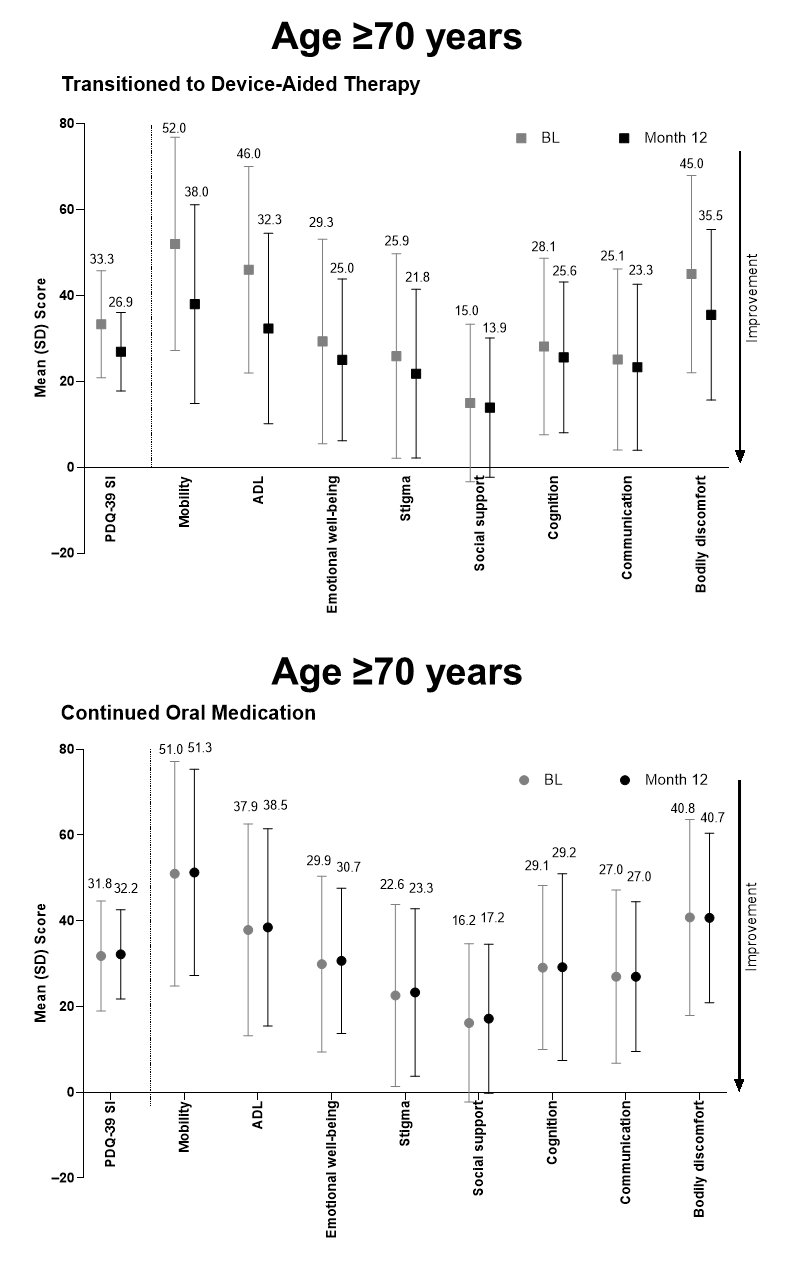

Supplement: Supplementary file 1 — Supplemental Material [file 41531_2025_1093_MOESM1_ESM.docx]
